# Association between the empirical dietary inflammatory index and cardiorespiratory fitness in Tehranian adults in 2017–2018

**DOI:** 10.3389/fnut.2022.928308

**Published:** 2022-09-21

**Authors:** Nastaran Payandeh, Hossein Shahinfar, Nadia Babaei, Samira Davarzani, Mojdeh Ebaditabar, Kurosh Djafarian, Sakineh Shab-Bidar

**Affiliations:** ^1^Department of Community Nutrition, School of Nutritional Sciences and Dietetics, Tehran University of Medical Sciences, Tehran, Iran; ^2^Department of Nutrition, School of Public Health, Iran University of Medical Sciences, Tehran, Iran; ^3^Department of Clinical Nutrition, School of Nutritional Sciences and Dietetics, Tehran University of Medical Sciences, Tehran, Iran

**Keywords:** empirically dietary inflammatory index, inflammation, cardiorespiratory fitness, VO2_max_, food-based dietary inflammatory index

## Abstract

**Background:**

Inflammatory-related chronic diseases are increasing in Iran with high consumption of a diet containing pro-inflammatory potential and a sedentary lifestyle. The empirical dietary inflammatory index (EDII) was developed as a tool to assess dietary effects on systemic inflammation. We examined the hypothesis that specific dietary patterns reflecting systemic inflammation are associated with cardiorespiratory fitness (CRF) in Tehranian adults.

**Methods:**

This cross-sectional study was carried out on 270 adults who are residents of Tehran. Dietary intake was assessed using a 168-item valid and reliable food frequency questionnaire. The EDII score was developed according to participant dietary intakes of 21-item pre-defined food groups. CRF was assessed by using a graded exercise treadmill test. Anthropometric measurements were assessed using standard methods. To discover the association between CRF and EDII, we used multivariable logistic regression analysis.

**Results:**

Those who were in the third tertile of the EDII had 57% lower odds of having better VO_2Max_ (ml/kg/min) than those in the first tertile (OR: 0.43; 95% CI: 0.16, 1.12, *p* = 0.01). There were no significant differences between tertiles of the EDII score in terms of VO2 (L·min) and VO2 (LBM) before and after adjusting for confounders. There was a significant decrease in VO_2Max_ (ml/kg/min) across tertiles of the EDII after controlling for covariates (*p*-value = 0.04). There was a significant inverse association between the EDII score and VO_2Max_ (ml/kg/min) (β = −0.35, *p* = 0.001).

**Conclusions:**

Our finding demonstrated that a higher EDII might be associated with lower CRF in Tehranian adults. Prospective studies are needed to shed light on the causal link between the EDII and CRF.

## Introduction

Chronic inflammation is a process in which inflammation develops slowly and lasts for a long period, ranging from months to years (1). An increase in inflammatory mediators such as C-reactive protein (CRP), tumor necrosis factor alpha (TNF-α), and interleukin 6 (IL6) is supposed to be associated with inflammation (2–4). Inflammation is a risk factor for chronic diseases such as cardiovascular diseases (CVDs), diabetes, and various cancers (5–7). Some research shows that cardiovascular diseases are one of the leading causes of mortality in adults older than 45 years (8). Also, there are numerous clinical investigations indicating that a strong association exists between low cardiorespiratory fitness (CRF) and the risk of mortality from cardiovascular disease (8–10).

CRF refers to the maximum capacity of the cardiovascular and respiratory systems to supply oxygen to skeletal muscles during activities such as exercise (10, 11). There are several studies available today which have examined the association between exercise and physical activity with inflammation, and their reports suggested that exercise has an anti-inflammatory role (12–14). Also, biological plausibility in the general population suggests that exercise has the potential to develop an acute and long-term anti-inflammatory phenotype (12, 14). The results of the study by Church et al. indicated that the level of CRF was inversely related to CRP levels in men (13).

According to studies, diet is one of the main determinants of inflammation (4, 15). Because of the interactions between food and micronutrients, the assessment of food intake is comprehensive when different food groups are examined together in the form of dietary patterns or food indexes. Dietary patterns or dietary indexes provide a more comprehensive assessment of an individual's diet than the approach of using single foods or nutrients to determine diet–disease associations (16, 17). It seems that the use of two different indicators (EDII and DII) to assess the inflammatory potential of the diet can cause differences in the results of different studies.

The two main indicators to describe the inflammatory potential of the diet include a literature-derived dietary inflammatory index (DII) and an empirical dietary inflammatory index (EDII) (15–18). The DII is essentially an *a priori* index focusing on the inflammatory potential of dietary nutrients (2), while the EDII is an *a posteriori* index focusing on the inflammatory potential of food groups, and this index appears to provide a new dimension of the inflammatory potential of the whole diet of individuals (15–17). According to the results of a meta-analysis of 17 related studies published in 2018, a higher DII increased the risk of CVD by about 35% and also showed a higher risk for all-cause CVD and cancer death (19). Recently, data from three large prospective cohort studies in the United States using the EDII showed that a higher incidence of CVD, CHD, and stroke was significantly associated with dietary patterns with higher inflammatory potential (20). On the other hand, Tabung et al. indicated that in contrast to the EDII, which is based on the food groups, the DII is a nutrient-based indicator and is strongly influenced by the consumption of nutritional supplements by individuals (15).

Given that Iran is a country with an increasing rate of several inflammatory diseases, we designed this cross-sectional study to investigate whether the inflammatory potential of diet (using the EDII to achieve a new dimension of results in the inflammation of food groups) is related to cardiorespiratory readiness in Tehranian adults.

## Methods

### Study population

This is a cross-sectional study in which 270 apparently healthy (118 males and 152 female) adults aged between 18 and 45 years living in Tehran from February 2017 to December 2018 with a mean age of 36.77 ± 13.19 years and an average BMI of 62.25 Kg/m^2^ were recruited by using the convenience sampling method. Based on the previously calculated correlation coefficient between the dietary pattern and cardiorespiratory fitness (21), our target number of subjects was 256 [($Z_{1-\frac{\alpha }{2}}+Z_{1-\beta }\times \sqrt{1-r^{2}}$ /*r*) = 256, where α = 0.05, 1–β = 0.95, and *r* = 0.34]. To compensate for the potential exclusion of participants due to under- and overreporting of total energy intake, or attrition due to other reasons, the final sample size of 270 participants was selected for inclusion. The inclusion criteria were age range of 18–45 years, being healthy, desire to participate in the study, and being residents of Tehran. Some people were excluded from the study who had a dietary intake of less than 800 kcal/d or more than 4200 kcal/d, infectious conditions, and active inflammatory diseases; who were pregnant and lactating; or who used drugs such as hormonal drugs or sedatives, as well as people who used any supplements for weight loss such as caffeine and green tea supplements and conjugated linoleic acid (CLA).

### Ethical considerations

In this study, written consent was obtained from all participants to participate in the study. Also, all methods were based on the Helsinki guideline and ethical standards of the Tehran University of Medical Sciences (TUMS.VCR.REC.1396.4085), which approved the protocol and the form of informed consent.

### Data collection

Participants' lifestyle information including age, gender, cardiovascular disease (yes or no), respiratory disease (yes or no), and smoking (yes or no) was collected through a self-administered questionnaire. Participants' physical activity was also evaluated using the Iranian version of the International Physical Activity Questionnaire (IPAQ) (22). Then, the participants were grouped into three categories: very low (< 600 MET-minute/week), low (600–3000 MET-minute/week), and moderate and high (> 3000 MET-minute/week) (23).

### Anthropometric measures and body composition

Body weight was measured in the participants while wearing light clothes to the nearest 0.1 kg by using a Seca scale. Participants' height was first measured by using a stadiometer (Seca, Germany), and their waist circumference (WC) was measured by placing a non-stretch tape measure in the middle of the last rib to just above the hipbones, and hip circumference (HC) was measured around the widest portion of the buttocks. By dividing the WC (cm) by the HC (cm), the waist-to-hip ratio (WHR) was obtained, and by dividing the weight (kg) by height (square meter), the body mass index (BMI) was calculated. The participants' body composition was assessed using InBody 720 (Biospace, Seoul, Korea). The participants were required to follow the following conditions before the measurements: not consume any foods for at least 4 hours, at least 2 liters of water consumption the day before, at least 8 h without physical activity, and at least 12 h without coffee or alcoholic beverages and also at least 24 h without using diuretics before the assessment.

### Cardiorespiratory fitness

CRF is an important test of physical fitness measurements which is related to the maximum oxygen consumption (VO2_Max_). According to the gold standard, VO2_Max_ is the best indicator of CRF and is measured by graded treadmill exercise tests. The participants were asked to accomplish a graded exercise protocol until exhaustion while they could breathe through a respiratory gas analyzer mask. The graded exercise test constitutes of seven steps with different treadmill settings, starting from 1.7 mph speed with 10% gradient. The speed and gradient of the treadmill are elevated every 3 minutes and the next step begins. The test was terminated when the subject was not able to keep exercising because of tiredness, pain, or any other medical symptom. Respiratory gases were analyzed by MetaLyzer3B during the test. Criteria used were as follows: (1) the respiratory exchange ratio and maximum heart rate reached 1.1 and (220_age) respectively. (2) The participant not to able to continue requested test interruption. CRF was measured with L/min, ml/min/kg body weight units. The resting metabolic rate (RMR) was measured by MetaLyzer3B and bioelectrical impedance analysis.

### Dietary assessment

Participants' dietary intakes were evaluated by trained nutritionists through face-to-face interviews using a valid, reliable, semi-quantitative 168-item food frequency questionnaire (FFQ) (24). This questionnaire reported the consumption of each food item on a daily, weekly, or monthly basis during the last year, and then these data were converted into daily consumption. Finally, all foods were analyzed in terms of their energy content using modified Nutritionist IV software prepared for Iranian food (version 7.0; *N*-Squared Computing, Salem, OR, USA).

We used the method introduced by Tubong et al. to calculate empirically derived daily and meal-based DII (EDII) (25). For this purpose, we used our dataset including 522 Tehranian people aged 18–70 years, in which foods and food groups contributing to systemic inflammation were explored. In that study, low-grade systemic inflammation was evaluated by measuring circulating high-sensitive C-reactive protein (hs-CRP) concentrations. Dietary intake was evaluated by using a food frequency questionnaire, and then foods were classified into 24 food groups. Major dietary patterns were identified through factor analysis, and accordingly, three major dietary patterns including Western, healthy, and traditional dietary patterns were identified. By applying multiple linear regression analysis, a positive association between the Western dietary pattern and levels of hs-CRP, as well as a negative association between healthy and traditional dietary patterns and hs-CRP concentrations was identified (data not shown). Thus, eight food groups contributing to the Western dietary patterns including eggs, snacks, nuts, mayonnaise, salt, processed meat, and fried potato were considered pro-inflammatory, and 16 food groups contributing to healthy and traditional dietary patterns including vegetables, canned fish, grains, olive, fruits, fishes, bread, high-fat dairy, low-fat dairy, legumes and soy, organ meat, boiled potato, oil and butter, coffee, pickles, and sweets and dessert were considered anti-inflammatory. Then, the average daily intakes of pro- and anti-inflammatory food groups for each participant were multiplied by their given factor loadings. The overall EDII score for each participant was computed by summing up the score of each 24 pro- and anti-inflammatory food groups. Finally, the EDII score was divided by 100 to reduce the magnitude of the score. A higher EDII score (more positive) indicates a more pro-inflammatory diet, and a lower EDII score (more negative) indicates a less pro-inflammatory diet.

### Statistical analysis

All the participants were grouped based on tertiles of the EDII. We used one-way ANOVA for quantitative data and chi-square tests for qualitative data, to compare general characteristics across EDII tertiles. The analysis of covariance (ANCOVA) was performed to assess the association between the EDII score with dietary intakes of the study participants after adjusting for potential confounders. The first tertile was considered the reference group. To evaluate the association between VO2_Max_, VO2 (L·min), and VO2 (LBM) across tertiles of the EDII, we used multiple linear regression analysis after adjustment for covariates such as age, sex, smoking, physical activity, body mass index, and energy intake. To find the association between the EDII with high/low CRF, we used multivariable logistic regression analysis. In the first model, we adjusted for energy intake, age, and sex. In the second model, we controlled for the confounding impact of more confounders such as age, sex, smoking, physical activity, cardiovascular disease, respiratory disease, and energy intake. In the last model, we adjusted all the variables adjusted in the second model along with the body mass index. The overall trend of odds ratios across tertiles of the EDII was calculated by considering the median of the EDII in each tertile as a continuous variable. All statistical analyses were performed with the Statistical Package for Social Sciences (SPSS) for Windows 25.0 software package (SPSS, Chicago, IL). The level of statistical significance was defined at *p* < 0.05.

## Results

General characteristics of the participants based on tertiles of the EDII are shown in Table 1. The participants' mean age was 36.7 ± 13.1 years, and their mean BMI was 25.6 ± 4.67. A total of 270 apparently healthy (117 male and 153 female) participants were included in our study. There was no significant difference among the mean of systolic blood pressure, smoking status, physical activity, CVD, and respiratory disease across the tertiles of the EDII. The mean of diastolic blood pressure, WC, weight, BMI, fat-free mass (F00FM), and fat mass (FM) showed a significant increase from the first tertile to the third tertile of EDII (*p* < 0.001 for all).

**Table 1:** General characteristic of participant according to the tertile of EDII.

		**EDII**		
		**T1** **89**	**T2** **90**	**T3** **89**
EDII score		(−1.32, 4.23)	(4.24, 6.82)	(6.83, 27.0)
Age (year)		35.4 ± 13.4	35.3 ± 12.7	39.0 ± 19.9
Blood pressure (mmHg)	Systolic	109 ± 16.6	109 ± 22.1	114 ± 18.0
	Diastolic	68.6 ± 10.7*	70.1 ± 11.2*	73.0 ± 9.69*
WC (cm)		86.1 ± 11.3*	88.3 ± 11.8*	94.3 ± 13.2*
Weight (Kg)		68.2 ± 14.7*	71.1 ± 15.0*	78.8 ± 16.7*
BMI (kg/m^2^)		24.5 ± 4.09*	25.4 ± 4.49*	26.9 ± 5.14*
FFM (kg)		47.7 ± 12.6*	48.3 ± 12.09*	54.4 ± 12.3*
FM (kg)		20.5 ± 8.45*	22.3 ± 8.87*	24.4 ± 10.5*
Sex, *n* (%)	Male	29 (24.8%)*	33 (28.2%)*	55 (47.0%)*
	Female	60 (39.7%)*	57 (37.7%)*	34 (22.5%)*
Smoking, *n* (%)	Yes	6 (30%)	5 (25%)	9 (45%)
	No	79 (34.1%)	82 (35.3%)	71 (30.6%)
	Quit	4 (28.6%)	1 (7.1%)	9(64.3%)
CVD, *n* (%)	Yes	1 (16.7%)	1 (16.7%)	4 (66.7%)
	No	88 (33.7%)	89 (34.1%)	84 (32.2%)
Respiratory disease, *n* (%)	Yes	0 (0.0%)	0 (0.0%)	3 (100%)
	No	89 (33.6%)	90 (34%)	86 (32.5%)
Physical activity, *n* (%)	Low	31 (30.4%)	42 (41.2%)	29 (28.4%)
	Moderate	38 (34.2%)	33 (29.7%)	40 (36%)
	High	20 (36.4%)	15 (27.3%)	20 (36.4%)

*p-*value < 0.05 was considered significant.

Significant *p*-value are showed with*.

Values are based on mean ± standard deviation or reported percentage.

One-way Anova for quantitative data and Chi-2 test for qualitative data have been used.

P_trend_ derived from polynomial regression test.

EDII, empirically dietary inflammatory index; WC, Waist circumference; BMI, body mass index; FFM, fat free mass; FM, fat mass; CVD, cardiovascular disease; WHR, waist to hip ratio; mmHg, millimeter of mercury; cm, centimeter; kg, kilogram; m, meter.

Subjects in the first tertile of EDII had EDII score between (−1.23, 4.23); second tertile: between (4.24, 6.82); third tertile: between (6.83, 27.0).

Table 2 shows the dietary intakes of the study participants according to tertiles of the EDII. The intake of refined grains, vegetables, soft drinks, mayonnaise, eggs, and pickles was higher in the top tertile of the EDII than in the first tertile. By contrast, the participants consumed fewer dairy products (high-fat and low-fat dairy), fish, fruits, and juices in the top tertile of the EDII than in the first tertile.

**Table 2:** Dietary intakes of the study participants according to the tertile of EDII.

	**T1**	**T2**	**T3**
Range	(−1.32, 4.23)	(4.24, 6.82)	(6.83, 27.0)
Subjects, n	89	90	89
Refined grains, g/d	255 ± 115*	344 ± 160*	511 ± 310*
Whole-grains, g/d	51.1 ± 50.3	95.5 ± 106	108 ± 116
Legumes, g/d	25.3 ± 25.2	25.5 ± 29.2	58.2 ± 73.8
Red or processed meat, g/d	32.7 ± 21.8	36.3 ± 35.0	59.0 ± 48.8
Vegetables, g/d	288 ± 165	321 ± 179	492 ± 350
Poultry, g/d	50.4 ± 85.7	50.7 ± 55.7	85.8 ± 165
Organ meat, g/d	2.81 ± 4.71	2.14 ± 3.47	5.37 ± 13.9
Vegetable Oils, g/d	6.24 ± 7.08	7.05 ± 5.96	7.68 ± 7.13
Soft drinks, g/d	28.5 ± 38.5*	39.5 ± 57.4*	81.1 ± 111*
Sweets and dessert, g/d	48.7 ± 35.6	59.3 ± 40.0	78.5 ± 47.6
Salt, g/d	5.69 ± 5.50	7.14 ± 6.05	7.43 ± 5.94
Mayonnaise, g/d	2.12 ± 3.17	2.17 ± 3.05	5.09 ± 9.77
Tea and coffee, g/d	378 ± 265*	633 ± 311*	1077 ± 618*
Salty snacks, g/d	6.98 ± 9.38	8.20 ± 11.8	11.4 ± 14.0
High-fat dairy, g/d	54.4 ± 58.5	75.6 ± 79.2	172 ± 235
French-fries, g/d	4.46 ± 5.86	5.69 ± 6.33	8.71 ± 10.2
Potato, g/d	19.0 ± 17.9	21.0 ± 17.9	25.9 ± 19.2
Low-fat dairy, g/d	472 ± 422*	317 ± 208*	351 ± 258*
Fruits and juices, g/d	339 ± 213	280 ± 166	313 ± 196
Nuts, g/d	11.0 ± 10.7	11.6 ± 14.7	14.0 ± 16.1
Fish, g/d	18.8 ± 21.2*	11.8 ± 10.4*	17.4 ± 18.6*
Egg, g/d	29.7 ± 41.6*	26.8 ± 31.1*	42.0 ± 52.7*
Pickles, g/d	6.94 ± 7.73	8.83 ± 10.7	19.1 ± 26.0
Hydrogenated fats, g/d	10.8 ± 13.3	13.8 ± 17.0	19.7 ± 23.5
Olive and olive oil, g/d	4.48 ± 4.99	4.50 ± 7.01	6.10 ± 7.62

All values were adjusted for energy intake using analysis of covariance.

Values are based on mean ± standard deviation.

*p*-value less than 0.05 was considered significant.

Significant *p*-value are showed with*.

EDII, empirically dietary inflammatory index; CRF, cardiorespiratory fitness.

Table 3 indicates the multivariate-adjusted means of CRF across tertiles of the EDII. In the crude model, we observed that there was a significant reduction in VO_2Max_ (ml/kg/min) across tertiles of the EDII. After adjusting for potential confounders (age, sex, smoking, physical activity, cardiovascular disease, respiratory disease, BMI, and energy), the relationship remained unchanged (*p*-value = 0.04). There was no significant difference in terms of VO_2Max_ (L·min) and VO_2Max_ (LBM) among tertiles of the EDII. After controlling for covariates, these associations remained non-significant.

**Table 3:** Multivariate adjusted means for CRF across tertiles of EDII.

	**Tertiles of EDII**					
	**T1 (−1.32, 4.23)**	**T2 (4.24, 6.82)**	**T3 (6.83, 27.0)**	***P*_1_**	***P*_2_**	***P*_3_**
VO_2_ Max (ml/kg/min)	31.8 ± 8.60	30.9 ± 7.14	29.0 ± 7.43	0.02	0.02	0.04
VO_2_ (L/min)	2.25 ±0.70	2.10 ± 0.65	2.35 ± 0.77	0.09	0.43	0.26
VO_2_ (LBM)	47.1 ± 8.99	46.8 ± 7.54	48.3 ± 8.39	0.49	0.34	0.81

*p*-value less than 0.05 was considered significant.

Values are based on mean ± standard deviation.

*P*_1_ derived from One-way analysis of variance.

*P*_2_ derived from polynomial regression test.

*P*_3_ derived from analysis of covariance.

Subjects in the first tertile of DII had DII score between (−1.32, 4.23); second tertile: between (4.24, 6.82); third tertile: between (6.83, 27.0).

EDII, empirically dietary inflammatory index; CRF, cardiorespiratory fitness.

P_*ANCOVA*_, Adjusted for age, sex, smoking, physical activity; CVD, respiratory disease, BMI and energy intake.

Multivariate adjusted odds ratios and 95% confidence intervals for having better CRF across tertiles of the EDII are presented in Table 4. In the crude model, although those who were in the third tertile of the EDII were less likely to have better VO_2Max_ (ml/kg/min) [OR= 0.81; CI95%: 0.39, 1.68] than those in the first tertile, there was no association between a higher EDII and VO_2Max_ (ml/kg/min) (*p* = 0.07) after adjusting for age, sex, smoking, physical activity, cardiovascular disease, respiratory disease, and energy intake; also, this result remained non-significant.

**Table 4:** Odds ratios (ORs) and 95% confidence intervals (95% CIs) for CRF according to tertiles of EDII.

	**Tertiles of EDII**			
	**T1**	**T2**	**T3**	
	**OR**	**OR (95%CI)**	**OR (95%CI)**	***P* trend**
**VO**_**2**_ **Max (ml/kg/min)**				
Crude	1	0.93(0.51, 1.68)	0.91(0.50, 1.65)	0.07
Model 1*	1	0.79(0.39, 1.60)	0.42(0.18, 1.00)	0.06
Model 2†	1	0.90(0.46, 2.2)	0.43(0.16, 1.12)	0.01
**VO**_**2**_ **(L/min)**				
Crude	1	0.68(0.37, 1.23)	0.95(0.53, 1.72)	0.35
Model 1	1	0.66(0.36, 1.20)	0.82(0.41, 1.63)	0.83
Model 2	1	0.64(0.35, 1.18)	0.87(0.43, 1.75)	0.96
**VO**_**2**_ **(LBM)**				
Crude	1	0.93(0.52, 1.68)	1.00(0.55, 1.80)	0.45
Model 1	1	0.88(0.48, 1.60)	0.82(0.41, 1.64)	0.96
Model 2	1	0.88(0.48, 1.61)	0.85(0.42, 1.72)	0.84

Data are OR (95%CI).

*Adjusted for age, sex, smoking, physical activity, Cardiovascular disease, respiratory disease, energy.

†Additionally adjusted for BMI.

EDII, Empirical dietary inflammatory index; LBM, Lean body mass; CRF Cardiorespiratory Fitness.

The linear associations between the EDII score and CRF are shown in Table 5. VO_2Max_ (ml/kg/min) had no significant linear association with EDII (β = −0.11, *p* = 0.33). Moreover, after controlling for covariates, these associations changed to significant (β = −0.35, *p* = 0.001).

**Table 5:** Multiple regression analysis models exploring the association of EDII with CRF.

	**β ± SE**	**95% CI**
**VO**_**2**_ **Max (ml/kg/min)**		
Model 1	−0.11 ± 0.12	−0.34, 0.11
Model 2	−0.35 ± 0.10*	−0.55, −0.14
**VO**_**2**_ **(L/min)**		
Model 1	0.01 ±0.01	−0.01, 0.03
Model 2	0.001 ± 0.01	−0.02, 0.03
**VO**_**2**_ **(LBM)**		
Model 1	0.09 ± 0.12	−0.15, 0.34
Model 2	0.03 ± 0.16	−0.30, 0.36

*p-*value < 0.05 was considered significant.

Significant *p*-value are showed with^*^.

Model 1 was crude.

Model 2 adjusted for age, sex, smoking, physical activity, *CVD*, respiratory diseases; BMI and energy intake.

EDII, empirically dietary inflammatory index; CRF, cardiorespiratory fitness; SE, standard error.

β coefficient obtained from linear regression.

## Discussion

Overall, we found that higher adherence to a diet containing higher pro-inflammatory potential may be associated with less Vo2_Max_ (ml/kg/min), and there is an inverse association between the EDII and Vo2_Max_ (ml/kg/min).

Cardiorespiratory fitness reflects the ability of cardiovascular and respiratory systems to transport oxygen to skeletal muscle mitochondria for energy production needed during physical activity (26). CRF is an important criterion for our mental and physical health and is affected by various factors such as age, genetics, body composition, gender, lifestyle, and the inflammatory potential of the diet (8, 26, 27). Recently, researchers have been using the EDII, instead of the DII, to measure the inflammatory potential of diet (20). The EDII is based on food groups, while the DII focuses on dietary nutrients (15).

Most available studies have used the nutrient-based DII score to assess the inflammatory potential of the diet, and they assessed the association of the DII score with various diseases, including cardiovascular disease (CVD), cancer, metabolic syndrome (Mets), and respiratory disease (2, 4, 28–30). According to the results of a prospective study, there was a positive association between the EDII and the risk of Mets in adults (16). They showed that a lower level of HDL-C and a higher level of FBG and WC were associated with a higher EDII score (16). They also indicated that there is no association between the EDII with hypertriglyceridemia and hypertension (16), while Camargo-Ramos et al. demonstrated that an increased inflammatory potential of the diet was inversely associated with an improved cardiometabolic profile, higher HDL-C, and lower Hb1Ac (31). In a 2017 study, Tabung et al. evaluated the validity of the EDII in two independent groups of men and women and found that the EDII significantly predicted the concentration of inflammatory markers (15). Also, the study of Li et al. showed that the EDII in large sample size has a positive relationship with inflammatory markers such as CRP, interleukin 6, and TNFα-R2 (20).

In a study by Wood et al., it was suggested that the Western diet may lead to systemic inflammation and subsequent inflammation of the airways and respiratory problems (32). By contrast, components of anti-inflammatory diets can have protective effects on the respiratory system in both adults and children (29, 33). Another case–control study by Wood et al. in 2015 on the adult population showed that the DII score was correlated with a systemic inflammation increase and less lung function. Each unit increase in the DII score can increase the risk of asthma by 70%. It also can reduce forced expiratory volume in 1 s by 3.44% (30).

On the contrary, some studies reported no association between the DII score and metabolic syndrome in young adults (34, 35). The results of a study by Christensen et al. in 2019 also showed a significant negative correlation among cardiorespiratory fitness, visceral fat, body fat, dietary fat, polyunsaturated and saturated fat, and CRP level (36). However, there was no association between the dietary inflammatory index and the CRP level in cancer survivors (36). Also, in a study by Asadi et al. on a middle-aged Iranian population, it was shown that there was no significant relationship between the DII and total cardiovascular disease, myocardial infarction, stable angina, or unstable angina (28).

The possible mechanisms by which an anti-inflammatory diet can reduce chronic disease or improve cardiorespiratory fitness are unclear. Despite this, it has been hypothesized that unhealthy dietary patterns with pro-inflammatory effects are associated with an innate immune response (increased production of pro-inflammatory cytokines and decreased production of anti-inflammatory cytokines). This eventually causes chronic inflammation and ultimately increases the risk of endothelial dysfunction, metabolic syndrome, cardiovascular disease, and cancer (27, 31).

Contradictory results of studies on the factors affecting CRF include different demographic characteristics of participants, other aspects of diet such as acidity and carcinogenic potential of the diet, different study design, different confounding factors, and the use of two different indicators to assess the inflammatory potential of the diet.

There are strengths in our study. The present study is the first Iranian study that examined the more complete EDII to evaluate the inflammatory potential of diet and its relationship with CRF and its components. We have used the valid 168-item FFQ that have been prepared to estimate the Iranian eating habits. In addition, we adjusted several important confounders, which could affect our results. Finally, using the EDII, instead of the DII, refers to the inflammatory potential of foods in the participants' diet. In addition, the use of an inflammatory index based on dietary groups can facilitate the recommendations of the anti-inflammatory diet by nutritionists. Also, unlike the DII, the EDII is not affected by the use of dietary supplements, which is a good advantage in clinical trials. However, the cross-sectional study we conducted has some limitations, which should be addressed in future studies. First, the study design was cross-sectional, which makes the conclusion of causality difficult. Second, we used the FFQ as a standard instrument to assess long-term dietary intake; however, estimates of food consumption from the FFQ are not precise, and there is always a probability of measurement error, although our FFQ was validated to have reasonably high validity (37). Third, the limited number of participants may have caused diminished statistical power in multivariable analyses.

Eventually, cardiorespiratory fitness appears to be affected by a variety of diet-related factors, including carcinogenicity of the diet and high acidity of the food (38–40). Although other dietary indicators, such as the alternate healthy eating index (AHEI), Dietary Approaches to Stop Hypertension (DASH), and Alternate Mediterranean Diet Score (AMED), assess the overall quality of the diet (41), the EDII specifically focuses on the potential of the diet to aid in chronic inflammation (15, 20). In this study, we only assessed the inflammatory aspect of the diet, but in future, more comprehensive studies with more detailed designs are needed.

## Conclusion

In summary, we found that a higher empirical DII is associated with increased VO2_Max_ after adjusting for confounding variables. The importance of following a healthy diet with a lower inflammatory index and having higher CRF should be considered as part of a healthy lifestyle approach, and longitudinal studies are needed to confirm our findings.

## Data availability statement

The original contributions presented in the study are included in the article/supplementary material, further inquiries can be directed to the corresponding author/s.

## Ethics statement

The studies involving human participants were reviewed and approved by Tehran University of Medical Sciences (Ethic Number: IR.TUMS.VCR.REC.1396.4085). The patients/participants provided their written informed consent to participate in this study.

## Author contributions

NP and SS-B contributed to conception and design of the research. ME, SD, and NB contributed to acquisition, analysis, or interpretation of the data. HS and NP drafted the manuscript. KD and SS-B critically revised the manuscript. SS-B agrees to be fully accountable for ensuring the integrity and accuracy of the work. All authors read and approved the final manuscript.

## Conflict of interest

The authors declare that the research was conducted in the absence of any commercial or financial relationships that could be construed as a potential conflict of interest.

## Publisher's note

All claims expressed in this article are solely those of the authors and do not necessarily represent those of their affiliated organizations, or those of the publisher, the editors and the reviewers. Any product that may be evaluated in this article, or claim that may be made by its manufacturer, is not guaranteed or endorsed by the publisher.
